# Book Reviews

**Published:** 1912-06

**Authors:** 


### BOOK REVIEWS

THE TAYLOR POCKET CASE RECORD. By J. J. Taylor, M. D., 252 pages, tough bond paper; red limp leather: $1.00. Published by The Medical Council Co., Forty-second and Chestnut Streets, Philadelphia, Pa.

The object of this book is to encourage more accurate observation and study of cases by supplying a convenient form for a condensed record of each important case, in pocket size, so that the practitioner can always have it with him, and so arranged that the necessary data can be written down in the briefest possible time—preferably while the examination is actually being made.

---

THE TREATMENT OF FRACTURES: With Notes upon a Few Common Dislocations. By Chas. L. Scudder, M. D., Surgeon to the Massachusetts General Hospital. Seventh Edition, Revised and Enlarged. Octavo volume of 708 pages, with 990 original illustrations. Philadelphia and London: W. B. Saunders Company, 1911. Polished Buckram, $6.00 net; half morocco, $7.50 net.

The seventh edition of this popular monograph differs from its predecessors in that it contains those facts which have been accepted during the past three years as important in the treatment of bone injuries. A special chapter is devoted to the operative treatment of fractures.

THE SURGICAL CLINICS OF JOHN B. MURPHY, M. D., at Mercy Hospital, Chicago. Volume I, Number I. Octavo of 133 pages, illustrated. Philadelphia and London: W. B. Saunders Company, 1912. Published Bi-Monthly. Price per year: Paper, $8.00; cloth, $12.00.

This is the first number of a series of books to be published every other month. Much valuable information is contained in this issue on 19 different surgical affections. Of course, one can not expect text-book descriptions, but instead more of the personal views of the author are given.

---

A MANUAL OF PATHOLOGY. By Guthrie McConnell, M. D., Professor of Pathology and Bacteriology, Temple University, Medical Dept., Philadelphia. Second revised Edition. 12mo of 531 pages, illustrated. Philadelphia and London: W. B. Saunders Company, 1911. Flexible Leather, $2.50 net.

McConnell takes up the subject of Pathology concisely in a clear and distinct manner, going into each subject thoroughly, yet not lengthy enough in theory to grow tiresome.

It enables the student to find at once what is most important, and gives the busy physician the cardinal principles of pathology at his finger tips.

---

A TEXT-BOOK OF PHYSIOLOGY: FOR MEDICAL STUDENTS AND PHYSICIANS. By William H. Howell, Ph. D., M. D., Professor of Physiology, Johns Hopkins University, Baltimore. Fourth Edition, Revised. Octavo of 1018 pages, fully illustrated. Philadelphia and London. W. B. Saunders Company, 1911. Cloth, $4.00 net; half morocco, $5.50 net.

Howell presents in this volume the important parts of physiology in a lucid and comprehensive manner. The present revision has added the necessary advances which keep it "in the current of the advancing tide of physiological knowledge."

BLAIR'S POCKET THERAPEUTICS: A Pratitioner's Handbook of Medical Treatment. By Thomas S. Blair, M. D., Neurologist to Harrisburg, Pa., Hospital; Author of "A System of Public Hygiene," "Blair's Practitioner's Handbook of Materia Medica," Member of the Harrisburg Academy of Medicine, American Medical Association, etc.; 373 pages, special Bible paper; bound in limp leather; price, $2.00. Published by The Medical Council Co., Forty-second and Chestnut Streets, Philadelphia, Pa.

---

PROGRESSIVE MEDICINE, A QUARTERLY DIGEST OF ADVANCES, DISCOVERIES AND IMPROVEMENTS IN THE MEDICAL AND SURGICAL SCIENCES. Edited by Hobart Amory Hare, M. D., Professor of Therapeutics and Materia Medica in the Jefferson Medical College of Philadelphia; Physician to the Jefferson Medical College Hospital; one time Clinical Professor of Diseases of Children in the University of Pennsylvania; Member of Association of American Physicians, Etc. Assisted by Leighton F. Appleman, M. D., Instructor in Therapeutics, Jefferson Medical College, Philadelphia; Ophthalmology, Philadelphia Polyclinic Hospital and College for Graduates in Medicine. Volume IV, December, 1911: Diseases of the Digestive Tracts and Allied Organs, the Liver, Pancreas and Peritoneum—Diseases of the Kidneys—Surgery of the Extremities, Shock, Anaesthesia, Infections, Fractures and Dislocations, and Tumors—Genito-Urinary Diseases—Practical Therapeutic Referendum. Lea & Febiger, Philadelphia.

Again we commend this excellent digest of medical and surgical advances.

